# Transcatheter aortic valve implantation with balloonexpandable valve: early experience from China

**DOI:** 10.5935/1678-9741.20150054

**Published:** 2015

**Authors:** Qingsheng Lu, Yifei Pei, Hong Wu, Zhinong Wang, Jing Zaiping

**Affiliations:** 1 Department of Vascular Surgery, Shanghai Changhai Hospital, China.; 2 Department of Cardiology, Shanghai Changhai Hospital, China.; 3 Department of Cardiothoracic Surgery, Shanghai Changzheng Hospital, China.; 4 Professor Shanghai Changhai Hospital, China.

**Keywords:** Aortic Valve Stenosis, Cardiac Catheterization, Heart Valve Diseases

## Abstract

**Objective:**

The aim of the current study was to evaluate the early experience of the application of transcatheter aortic valve implantation with the balloon-expandable system in China. The transcatheter aortic valve implantation technology has been widely used for patients with inoperable severe aortic stenosis in the developed world. The application of transcatheter aortic valve implantation is still in the early stages of testing in China, particularly for the balloon-expandable valve procedure.

**Methods:**

This was a retrospective study. All patients undergoing transcatheter aortic valve implantation with balloon-expandable system in our hospital between 2011 and 2014 were included. Edwards SAPIEN XT Transcatheter Heart Valve was used. The improvement of valve and heart function was evaluated as well as 30-day mortality and major complications according to the VARC-2 definition.

**Results:**

A total of 10 transcatheter aortic valve implantation procedures with the balloon-expandable system were performed in our hospital, of which 9 were transfemoral and 1 was transapical. The median age was 76 years, and the median STS score and Logistic EuroSCORE (%) were 8.9 and 16.2. The implantation was successfully conducted in all patients, only 2 patients had mild paravalvular leak. There was no second valve implantation. Moreover, no 30-day mortality or complications was reported. Following the transcatheter aortic valve implantation procedure, the heart and valve functions had improved significantly. During the follow-up period of 3-34 months, one patient died of lung cancer 13 months after the operation.

**Conclusion:**

This early experience has provided preliminary evidence for the safety and efficacy of transcatheter aortic valve implantation procedure with the balloon-expandable system in the developing world with an increasing aging population.

## Abstract (pt)

**Objetivo:**

O objetivo do presente estudo foi avaliar a experiência inicial da aplicação do implante percutâneo da válvula aórtica com o sistema balão-expansível na China. A tecnologia TAVI tem sido amplamente utilizada para pacientes com estenose aórtica grave inoperável no mundo desenvolvido. A aplicação de implante percutâneo da válvula aórtica está ainda nas etapas iniciais de teste na China, em particular o procedimento de válvula balão-expansível.

**Métodos:**

O estudo foi retrospectivo e todos os pacientes submetidos a implante percutâneo da válvula aórtica com sistema balão-expansível em nosso hospital entre 2011 e 2014 foram incluídos. Edwards SAPIEN XT Válvula Cardíaca Transcatheter foi usado. A melhoria do funcionamento da válvula e do coração foi avaliada, bem como mortalidade em 30 dias e as principais complicações de acordo com a definição VARC-2.

**Resultados:**

Um total de 10 procedimentos Tavi com o sistema balão-expansível foram realizados em nosso hospital, dos quais 9 foram transfemorais e 1 foi transapical. A idade média foi de 76 anos, e os STS mediana marcar e Logistic EuroSCORE (%) foram de 8,9 e 16,2. A implantação foi realizada com sucesso em todos os pacientes, apenas 2 pacientes tiveram vazamento paravalvar leve. Não houve um futuro implante valvar. Além disso, mortalidade em 30 dias ou complicações não foram relatadas. Seguindo o procedimento de implante percutâneo da válvula aórtica, as válvulas cardíacas e funções melhoraram significativamente. Durante o período de acompanhamento de 3-34 meses, um paciente morreu de câncer de pulmão 13 meses após a operação.

**Conclusão:**

Esta experiência inicial apresentou elementos de prova preliminar para a segurança e eficácia do procedimento implante percutâneo da válvula aórtica com o sistema balão-expansível no mundo em desenvolvimento com crescente envelhecimento da população.

**Table:** 

**Abbreviations, acronyms & symbols**	
AS	Aortic stenosis
EOAI	Effective disc mouth area index
LVEF	Left ventricular ejection fraction
NYHA	New York Heart Association
PARTNER	Placement of aortic transcatheter valves
SAVR	Surgical aortic valve replacements
SD	Standard deviation
TAVR	Transcatheter aortic valve replacement
TTE	Transthoracic echocardiography
TAVI	Transcatheter aortic valve implantation

## INTRODUCTION

During the past 50 years, the etiology of valvular heart diseases has changed greatly in developed countries, with an increase in non-rheumatic valvular heart diseases such as age-related calcific aortic stenosis (AS)^[1,2]^. AS is now considered one of the most common valvular diseases in the developed world. For instance, the year-round surgical aortic valve replacements (SAVR) quantity is estimated to be 67,500 in the United States^[3]^. In China, although limited data indicated that the prevalence of rheumatic heart disease was 10 times higher than developed countries in 2002^[4]^, the rapid growth of an aging population also increases the number of vulnerable age-related AS.

Once AS becomes severe and symptomatic, the prognosis is poor with high mortality if left untreated^[5,6]^. A recent meta-analysis found 69% and 36% of increased risks of cardiovascular and consequential mortality in AS patients, respectively^[7]^, might be partially explained by the selected high risk patients with older age and comorbities. Although the conventional SAVR has excellent outcomes^[8-10]^, it has been reported that patients with severe symptomatic AS had higher mortality when treated by SAVR^[11,12]^. The emergence and rapid development of transcatheter aortic valve replacement (TAVR) indicated hope for those inoperable or high-risk patients^[13,14]^. Since 2007, more than 100,000 patients have been treated by TAVR worldwide^[15]^, most of whom were from developed countries. Moreover, a recent meta-analysis estimated that approximately 290,000 elderly patients are TAVR candidates in European countries and North America^[16]^. Within the 2 widely used device types, the use of balloon-expandable valve (Cribier-Edward) has been shown to have higher success rate than self-expandable valve (CoreValve) in a multi-center study^[17]^. However, evidence from developing countries was scarce. For example, the initial experience of transcatheter aortic valve implantation (TAVI) was reported in Brazil^[18,19]^, South Africa^[20]^, and India^[21]^, respectively. In mainland China, the use of TAVI did not start until the first successful procedure with self-expandable valve in 2010^[22]^. The use of balloon-expandable valve remains limited.

Since 2011, our hospital was the first to introduce TAVI with the balloon-expandable system in mainland China. The present study aimed to evaluate the early experience of TAVI procedure using balloon-expandable valve in mainland China, and to provide potential evidence for the application and generalization of this novel technology in the developing world with an increasing aging population.

## METHODS

### Patients

This was a retrospective study. All patients that underwent TAVI with the balloon-expandable system in our hospital between 2011 and 2014 were included. All patients were selected by a multidisciplinary core team after extensive screening, including transthoracic echocardiography, coronary arteriography, computed tomographic angiography and lung function examination to evaluate the severity of AS and the existence of any contraindications. Patients that met at least one of the following criteria were included: 1) severe AS with an aortic valve area < 1 cm^2^; 2) a New York Heart Association (NYHA) functional class II or higher; 3) a STS of 5%~15%; or 4) a Logistic EuroSCORE of 20% or higher. Exclusion criteria included bicuspid aortic valve, acute myocardial infarction, LVEF<20%, aortic valve ring> 25mm or <18mm, severe coronary artery diseases, severe aortic or mitral regurgitation, severe kidney dysfunction, or transient ischemic attack within 6 month. Eligible patients had aortic annulus diameters of 20-25 mm, as determined by the transesophageal echocardiography (TEE). A total of 10 patients (9 male) with NYHA functional class II or higher were included in the current study.

### Ethics Statement

The current study was approved by the Research Ethics Committee of Shanghai Changhai Hospital (CHEC2011-099, 9/16/2011) and a waiver of informed consents was granted as the data were retrospectively reviewed and analyzed anonymously.

### Device and procedure

**Fig. 1:**
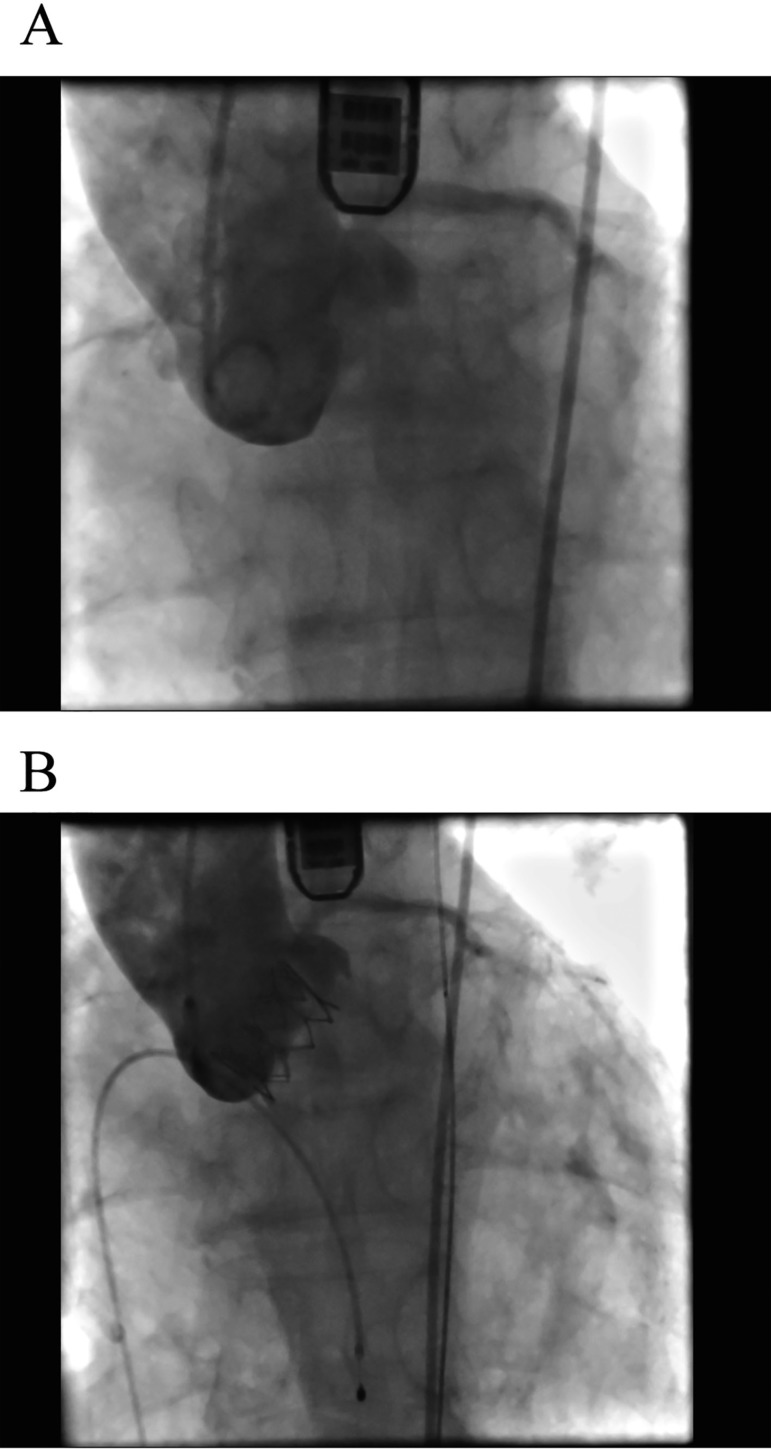
Angiography image before (A) and after (B) balloonexpandable valve implantation.

Procedures were performed in hybrid operating room under intratracheal intubation anesthesia. Edwards SAPIEN XT Transcatheter Heart Valve (Edwards Lifescience Corp) was used for all patients (23 and 26 mm). Aortic valve multidetector computed tomography and aorta computed tomography angiography were done before the procedure to evaluate the calcification level. Biplanar TEE was used for real-time supervision during the TAVI procedure. The aortic annulus diameter, aortic valve area, regurgitation velocity, regurgitation gradient, and distance from coronary artery were reevaluated by TEE. A standard transfemoral retrograde approach was applied. In brief, a 18-F eSheath with the introducer (5Fr sheath) was inserted over a 0.035 guidewire into the femoral artery. The 5Fr sheath was advanced over an extra stiff 0.035-inch guide wire (Amplatz, Cook, Inc., Bloomington, IN) into the left ventricle. The 5Fr sheath was then retrieved and the eSheath was introduced to the aortic valve. Following the balloon aortic valvuloplasty under rapid ventricular pacing, the SAPIEN XT Transcatheter Heart Valve delivery system was inserted into the eSheath hub. After accurate positioning by aortic root angiograms and TEE guidance, the Transcatheter Heart Valve was deployed during rapid ventricular pacing (Figure 1). Thereafter, the delivery system was retrieved and the femoral access site was percutaneously closed (ProGlide^TM^, Abbott Vascular, Inc., Abbott Park, IL). For cases that failed to deliver the SAPIEN XT Transcatheter Heart Valve through the femoral access, a transapical approach was conducted through the left ventricular apex. The transpical approacch was previous planned since the patient had severe iliac artery calcification.

### Outcome measurements

The indicators of valve and heart functions including: left ventricular ejection fraction (LVEF, %); aortic annulus diameter (mm); aortic valve area (cm^2^); effective disc mouth area index (EOAI) and jet velocity (cm/s) were assessed. In regards to the prognosis indicators, the operative (30-day) mortality and major complications including: cerebrovascular accident; arrhythmia; congestive heart failure; myocardial infarction; angina; paravalvular regurgitation; valve migration; valve infection; bleeding; pulmonary infection; urinary system infection; respiratory failure and dialysis-dependent renal failure were evaluated. Outcomes were also evaluated by the VARC II definitions.

### Statistical analyses

Data were presented as median (IQR) for continuous variables, and numbers (%) for categorical variables. The comparison of valve and heart function before and after TVAR were analyzed by Wilcoxon signed-rank test or Fisher's Exact Test. A *P* value of *P*<0.05 was considered statistically significant.

The funding sources had no role in study design; in the collection, analysis, and interpretation of data; in manuscript writing; or in the decision to submit the article for publication.

## RESULTS

Between 2011 and 2014, a total of 10 patients (9 male) underwent TAVI procedures with the balloon-expandable system in our hospital, of which 9 were transfemoral and 1 was transapical. The baseline and procedure characteristics of patients were presented in Table 1. The median age was 76 (IQR: 75, 78) years. All patients had a NYHA functional class II or higher. Three patients (30%) had atrioventricular block. The median STS score and Logistic EuroSCORE (%) were 8.9 (IQR: 8.1, 11.0) and 16.2 (IQR: 15.5, 17.7), respectively.

**Table 1:** Baseline and Procedure Characteristics (n=10).

Patient Characteristics	
Baseline Characteristics	
Age (years)*	76 (75,78)
Gender, male, n (%)	9 (90.0)
Hypertension, yes, n (%)	9 (90.0)
Diabetes, yes, n (%)	2 (20.0)
Coronary artery stenosis, yes, n (%)	3 (30.0)
Surgery history, yes, n (%)	4 (40.0)
Other comorbidities, yes, n (%)	2 (20.0)
Aortic calcification, yes, n (%)	
Mildly calcified	6 (60.0)
Multiple spots	3 (30.0)
Iliac artery stenosis	1 (10.0)
Femoral artery diameter, yes, n (%)	
7.0-8.0 mm	4 (40.0)
8.1-9.0 mm	5 (50.0)
>9.0 mm	1 (10.0)
NYHA functional class, yes, n (%)	
I	0
≥ II	10 (100)
Atrioventricular block, yes, n (%)	3 (30.0)
STS score*	8.9 (8.1, 11.0)
LVEF (%)*	62.6 (55.0, 67.0)
TTE Aortic annulus diameter (mm)*	23.0 (21.0, 24.0)
TTE Aortic valve area (cm^2^)*	0.86 (0.82, 0.90)
EOAI*	0.52 (0.49, 0.53)
Peak trans aortic gradient (mmHg)*	104 (88, 117)
Jet velocity (cm/s)*	510 (456,542)
Logistic EuroSCORE (%)*	16.2 (15.5, 17.7)
Procedure Characteristics	
Procedure time (min)*	200 (185, 255)
Blood products (ml)*	200 (0, 800)
Length of stay (day)*	7 (6, 8)
ICU time (day)*	0 (0, 1)
Mild paravalvular leak, yes, n (%)	2 (20.0)
Readmission, yes, n (%)	0 (0)

* Data were presented as median (IQR) for continuous variables.

EOAI=effective disc mouth area index; NYHA=New York Heart Association; LVEF=left ventricular ejection fraction; TTE=transthoracic echocardiography

Following successful implantations with a median procedure time of 200 min, all patients had stable vital signs and were discharged from hospital 4 to 8 days after the TAVI procedure. Only 2 patients (20%) had mild paravalvular leak (Table 1). There was no second valve implantation, valve migration, or infection in any patient. In addition, there was no 30-day mortality or vascular complications according to the VARC-2 definition (Table 2). During the follow-up period of 3-34 months, 1 patient died of lung cancer at 13 months after the TAVI procedure.

**Table 2:** The VARC-2 outcomes in the 30-day follow up period.

Outcomes	Number (n=10)
All-cause mortality	0
Cardiac mortality	0
Stroke	0
Life-threatening bleeding	0
Acute kidney injury, stage 2 or 3	0
Coronary artery obstruction	0
Major vascular complication	0
Valve-related dysfunction requiring repeat procedure	0

Following the TAVI procedure, the valve functions were significantly improved (Table 3). The median aortic annulus diameter, aortic valve area and EOAI were significantly increased from 23.0 (IQR: 21.0, 24.0) mm to 26.0 (IQR: 23.0, 26.0) mm, 0.86 (IQR: 0.82, 0.90) cm^2^ to 1.78 (IQR: 1.72, 1.80) cm^2^ and 0.52 (IQR: 0.49, 0.53) to 1.06 (IQR: 1.01, 1.12), respectively (all *P*<0.05). In addition, the median jet velocity decreased from 510 (IQR: 456, 542) to 205 (IQR: 186, 236) cm/s (*P*<0.001). Meanwhile, the NYHA functional class of 8 patients (80%) had improved to class I within 30 days in the postoperative period.

**Table 3:** Comparison of valve and heart function before and after TAVI.

Baseline Characteristics	Before TAVI	After TAVI	*P* value
LVEF(%)	62.6 (55.0, 67.0)	64.5 (61.0, 67.0)	0.43
TTE Aortic annulus diameter (mm)	23.0 (21.0, 24.0)	26.0 (23.0, 26.0)	0.021
TTE Aortic valve area (cm^2^)	0.86 (0.82, 0.90)	1.78 (1.72, 1.80)	<0.001
EOAI	0.52 (0.49, 0.53)	1.06 (1.01, 1.12)	<0.001
Jet velocity (cm/s)	510 (456, 542)	205 (186, 236)	<0.001
NYHA functional class ≥ II, yes, n (%)	10 (100)	2 (20)	<0.001

Data were presented as median (IQR) or n (%).

EOAI=effective disc mouth area index; NYHA=New York Heart Association; LVEF=left ventricular ejection fraction; TTE=transthoracic echocardiography

## DISCUSSION

According to the China Report of the Development on Aging Cause, the percentage of the aging population in China was 14.8% (more than 0.2 billion) in 2013^[23]^. The rapid growth of an aging population has resulted in a significant challenge in defending age-related chronic diseases, such as calcific AS. Due to poor prognosis after the manifestation of cardiovascular symptoms, safe and effective medical procedures are in urgent need to treat AS, particularly for those elderly inoperable patients. Our hospital was the first to introduce TAVI with the balloon-expandable system in mainland China. Our early experiences with favorable improvement of heart and valve function has provided preliminary evidence for the safety and efficacy of the TAVI procedure in severe AS patients in mainland China.

Although conventional SAVR has excellent outcomes^[8-10]^, the mortality and morbidity rates remain high in patients at extreme high-risk or inoperable patients with severe AS^[11,12]^. The emergence of TAVR technology offers a novel, less-invasive approach with a success procedure rate of over 93%^[24-26]^. The advantages of TAVR procedure have been evaluated thoroughly in developed counties, especially from large-scale studies of registry data from US and European countries including more than 10,000 patients^[27-29]^. Moreover, according to the findings from the Placement of Aortic Transcatheter Valves (PARTNER) B trial, inoperable patients that underwent TAVR had significantly lower 1-year mortality rate compared with those patients that underwent standard therapy (30.7% vs. 50.7%, respectively). Among the survivors, the 1-year rate of cardiac symptoms was also lower in the TAVR group compared with the standard therapy group (25.2% vs 58.0%, respectively)^[30]^. In the PARTNER A trial, the TAVR and SAVR procedure had comparable mortality and symptom improvement for high-risk surgical candidates during the 2-year follow-up period^[31,32]^. However, evidence from developing countries including China remains limited.

Mortality, as well as major complications such as cerebrovascular accident, paravalvular regurgitation, and vascular events, are concerning implications for the use of the TAVR procedure. In the TAVR group of the PARTNER A trial, the 30-day mortality, major cerebrovascular accident, and major vascular complications were 3.4%, 3.8% and 11.0%, respectively^[31]^, whilst the all-cause mortality 2 years after the TAVR procedure was 33.9%^[32]^. Moreover, among all eligible TAVR cases utilizing the Sapien Transcatheter Heart Valve from November 2011 to May 2013 in the United States, the in-hospital mortality and cerebrovascular accident rates were 5.5% and 2.0%, respectively^[33]^. In contrast, in the present study, there were zero cases of mortality and major complications in the 30-day follow-up period and only one patient died of lung cancer 13 months after the TAVR procedure during the follow-up period. Although a decisive conclusion of low mortality and complication rate could not be made based on the findings of the current study, the extensive screening and careful evaluation for all patients by a multidisciplinary core team before TAVR procedure may have contributed to the higher success rate and better prognosis. In addition, in terms of the incorporation of TAVR in clinical practice, functional improvement would provide valuable information. A systematic review of current reports revealed consistent benefits of TAVR by the improvement in NYHA functional class^[34]^. In the current study, improvement in heart and valve function after TAVI was also observed. Studies with a larger number of cases and a longer follow-up period are required to validate the findings of the current study.

Currently, the TAVR approach has been widely utilized in developed countries. The application of TAVR is, however still in the very early stages in developing countries such as China, due to high-demand technology and expensive therapeutic fees. Whilst previous reports have shown favorable outcomes of the TAVR procedure in inoperable or high-risk patients with severe AS, the generalization of TAVR in routine therapy remains complex, including the accessibility of a facility for this procedure in a clinical center, the experience of the operator and the core team, the selection and evaluation of high-risk patients, the procedure performance, and perioperative and postoperative care. Furthermore, whilst rapid incorporation of the TAVR procedure in clinical treatment is progressing, technical challenges remain^[15]^. In the PARTNER A trial, among patients that have undergone TAVR, there was increased paravalvular regurgitation and major vascular complications than those patients treated by SAVR^[31,32]^. In the PARTNER B trial, there was also a higher incidence of major cerebrovascular accident and major vascular events in the TAVR group compared with the standard therapy group^[30]^. In contrast, the considerably advanced technology in the SAVR procedure has greatly improved the surgical results in high-risk patients^[35]^. To avoid the misuse of TAVR, the ACCF/AATS/SCAI/STS expert consensus published in 2012, has provided standards for applying TAVR in the clinical practice in the United States^[5]^. However, whether these standards derived from Western populations, can be applied or not in China, remain unknown. Moreover, whether the commercially available Transcatheter Heart Valve designed for Western patients will fit Chinese patients needs to be elucidated in large multicenter studies with longer follow-up duration.

### Strength and Limitations

To the best of our knowledge, the current study was the first attempt to evaluate the TAVI approach with the balloon-expandable system in mainland China. This study contains several limitations. The number of patients that underwent the TAVI procedure with the balloon-expandable system was very small, due to the demanding technology and high expenditure of this procedure. Secondly, the current study was a single center study. Due to the complexity of incorporating TAVI in clinical practice, the results of the current study may not be generalized to other centers in China.

## CONCLUSION

In conclusion, the current study has provided an evaluation of early experience in the application of TAVI procedure with the balloon-expandable system in mainland China. Further clinical evidence and longer follow-up duration are required to further explore the clinical value and general possibility of introducing TAVI procedure in the developing world with an increasing aging population.

**Table:** 

**Authors’ roles & responsibilities**	
QL	Analysis and/or interpretation of data; statistical analysis; final manuscript approval; implementation of projects and or experiments; manuscript writing or critical review of its content
YP	Final manuscript approval; implementation of projects and/ or experiments; manuscript writing or critical review of its content
HW	Final manuscript approval; implementation of projects and/ or experiments; manuscript writing or critical review of its content
ZW	Final manuscript approval; implementation of projects and/ or experiments; manuscript writing or critical review of its content
JZ	Final manuscript approval; study design; implementation of projects and/or experiments; manuscript writing or critical review of its content
